# Long-term trends in seasonality of mortality in urban Madagascar: the role of the epidemiological transition

**DOI:** 10.1080/16549716.2020.1717411

**Published:** 2020-02-06

**Authors:** Benjamin-Samuel Schlüter, Bruno Masquelier, C. Jessica E. Metcalf, Anjarasoa Rasoanomenjanahary

**Affiliations:** a Center for Demographic Research (DEMO), Université Catholique De Louvain (UCL), Louvain-la-Neuve, Belgium; b French Institute for Demographic Studies, Paris, France; c Department of Ecology and Evolutionary Biology and the Woodrow Wilson School, Princeton University, Princeton, NJ, USA; d Bureau Municipal d’Hygiène De La Commune Urbaine d’Antananarivo, Antananarivo, Madagascar

**Keywords:** Seasonality of mortality, changes in seasonal patterns, epidemiological transition, Madagascar

## Abstract

**Background:** Seasonal patterns of mortality have been identified in Sub-Saharan Africa but their changes over time are not well documented.

**Objective:** Based on death notification data from Antananarivo, the capital city of Madagascar, this study assesses seasonal patterns of all-cause and cause-specific mortality by age groups and evaluates how these patterns changed over the period 1976–2015.

**Methods:** Monthly numbers of deaths by cause were obtained from death registers maintained by the Municipal Hygiene Office in charge of verifying deaths before the issuance of burial permits. Generalized Additive Mixed regression models (GAMM) were used to test for seasonality in mortality and its changes over the last four decades, controlling for long-term trends in mortality.

**Results:** Among children, risks of dying were the highest during the hot and rainy season, but seasonality in child mortality has significantly declined since the mid-1970s, as a result of declines in the burden of infectious diseases and nutritional deficiencies. In adults aged 60 and above, all-cause mortality rates are the highest in the dry and cold season, due to peaks in cardiovascular diseases, with little change over time. Overall, changes in the seasonality of all-cause mortality have been driven by shifts in the hierarchy of causes of death, while changes in the seasonality within broad categories of causes of death have been modest.

**Conclusion**: Shifts in disease patterns brought about by the epidemiological transition, rather than changes in seasonal variation in cause-specific mortality, are the main drivers of trends in the seasonality of all-cause mortality.

## Background

In many parts of the world, deaths exhibit strong seasonal variation, especially among children and the elderly. The underlying drivers may be varied. Many aspects of human biology relevant to health status are seasonal. For example, vitamin D metabolism and sunlight have been suggested as important drivers of seasonality in immune function [1]. Food intake can also vary substantially seasonally, from fluctuations in access to fruit and vegetables through to the emergence of a ‘hungry season’ in the most severe cases, where poor rural families are unable to maintain body weight and function throughout the year [2]. Both non-infectious and infectious causes of mortality will be modulated by such underlying biology. For non-infectious causes of mortality, seasonal fluctuations in temperature may modulate associated risk factors (such as the effects of temperature on stroke [3,4]); and seasonal fluctuations in behavior may alter psychological conditions (e.g. depression), or exposure to pollutants [5]. For infectious diseases, climatic variables may drive additional seasonality for a range of pathogens, via their effects on vector life-cycles, how infectious particles fall out of the air for directly transmitted pathogens or by how flooding shapes transmission of water-borne infections [6]. Seasonal patterns of human behavior have also been shown to be a key driver of infections, with seasonal aggregation due to school terms [7] or seasonal migration [8] increasing the magnitude of measles transmission.

The drivers of seasonal variation in mortality are also subject to change over time, due to (i) improvements in socioeconomic conditions, (ii) epidemiological shifts, and (iii) the effects of climate change. All three might directly alter the dominant causes of death or shift their distribution over the course of the year. Taking each in turn, first, in western countries, there is some evidence of a reduction in the seasonality of mortality, partly because of the spread of central heating and improvements in housing [9–11]. Second, the epidemiological transition has led to a reorganization of the hierarchy of causes of death. This can drive trends in the seasonality in mortality because seasonal variation is larger for some diseases than for others. For example, seasonal variation is characteristics of many infectious and parasitic diseases (including malaria), cardiovascular diseases, respiratory diseases, and acute gastroenteritis [12,13], but rare for neonatal disorders and neoplasms [14,15]. Third, climate change can also affect the seasonality of mortality, e.g. via increases in mortality in the summertime due to the increased frequency of heatwaves [16] or effects on pathogen life history [6].

With few exceptions [12,13,17,18], the literature on seasonal variation in mortality in Sub-Saharan Africa is patchy, especially when it comes to analyzing cause-specific mortality or long-term changes. This is because analyzing seasonal patterns requires statistical series of deaths tabulated by months, for which death registers are the preferred source of data. However, few countries in Sub-Saharan Africa have a comprehensive system of death registration in place. Often less than half of all deaths are registered, and causes of death are rarely established [19]. As a result, few countries in Sub-Saharan Africa have high-quality data on causes of death, apart from geographically defined populations monitored in Health and Demographic Surveillance Systems (HDSS). Some studies based on HDSS data in Africa have highlighted strong associations between temperature or rainfall and all-cause mortality but they were limited by the relatively short length of the periods covered, the absence of disaggregation by cause of death and their concentration in rural areas [12,13,17,18,20–22].

In Antananarivo, the capital city of Madagascar, a unique data source provides the opportunity to examine seasonality in cause-specific mortality over a long period. The notification of deaths to the health system was introduced in 1921 in the Municipal Hygiene Office (henceforth BMH for Bureau Municipal d’Hygiène), in response to a plague epidemic. The BMH issues a death verification form that is required to obtain a burial permit or to move the body outside the city to reach the family tomb. All records covering the period from 1976 to 2015 were transcribed from registers maintained by the BMH. Previous research has shown that this death notification system can be considered complete since the mid-1970s [23,24].

In this study, we capture the seasonal patterns of mortality for infants, children aged 1–4, older children and adults aged 5–59 years, and the population aged 60 and above. We evaluate whether these seasonal patterns have changed over a 40-year period. We hypothesize that the seasonality of deaths in childhood has attenuated because the share of communicable diseases has reduced, and the burden of neonatal disorders has increased. Based on the literature related to high-income countries, we anticipate a reduction in the seasonality of mortality among older adults. We also examine whether changes in seasonal variation can be ascribed to changes in the seasonality of cause-specific mortality or shifts in the hierarchy of causes of death.

## Methods

### Setting

**Figure 1.**
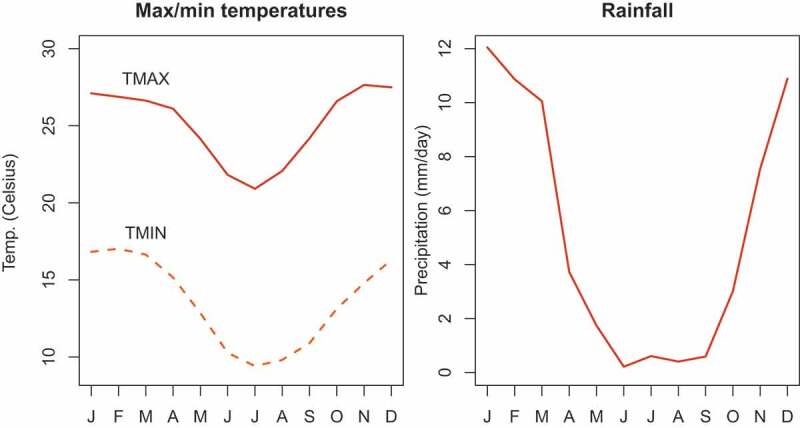
Monthly means of daily maximum/minimum temperatures and rainfall in Ivato station (over the period 1976–2015). Sc: DGHCN/daily

Antananarivo is located in the central highlands of Madagascar and culminates at an altitude of 1280 m. It has a subtropical climate with a cold and dry season from May to October (with average minimal temperature around 11°C) and a hot and rainy season from November to April (with average maximal temperature around 27°C) (Figure 1). December, January and February are the 3 months with the highest rainfall and they also correspond to the lean season. Rice is by far most consumed staple food in Madagascar; it furnishes more than 50% of the average calorie ration of the country. The cropping calendar varies greatly according to rice species and climate conditions of the regions, but about 70% of the rice produced in the country is harvested between April and June. Because of its predominance in the agriculture and diet, the seasonal production of rice drives seasonal movements in food prices and overall food consumption [25].

Since the 1950s, climate change has led to a rise in temperatures in Madagascar, particularly in the dry season [26]. The rainy season is also being delayed [27]. The frequency of extreme events such as cyclones, floods and droughts is increasing. All these changes are likely to modify seasonal patterns of mortality.

Antananarivo has undergone a major epidemiological transition in the last decades, only interrupted by a mortality crisis in the mid-1980s caused by the combination of the resurgence of malaria and food shortages [28,29]. Life expectancy first declined from 56 in 1976 to 47 in 1986, before increasing steadily to reach 64 years in 2015 (Figure S1). This progress was mostly driven by a decline in under-five mortality, which fell to 34 deaths per thousand live births in 2015, from 116‰ in 1976 (a 70% decline). By comparison, there has been virtually no improvement in survival changes in adults: the risk of dying between ages 15 and 60 was 280 deaths per thousand in 1976, peaked at 473‰ in 1986, at the height of the crisis, and declined again to 283‰ in 2015. The contrast between child and adult mortality suggests that survival gains were for the most part achieved through public health interventions targeting diarrheal and vaccine-preventable diseases. Demographic and Health Surveys show increases in the percentage of children who received all 8 basic vaccinations (BCG, DPT1-3, Polio 1–3 and measles), from 43% in 1992 to 62% in 2008–2009 in the country [30]. Chronic malnutrition has been slightly reduced; the prevalence of stunting in children under age 5 declined from 60% in 1992 to 50% in 2008–2009 (47% in the capital). In contrast, there has been little progress in skilled attendance at birth, access to improved water sources and sanitation. According to the 2008–2009 DHS, only 14% of the population of the capital lived in households with improved, non-shared toilet facilities [30]. Seventy percent of the population had access to water from public taps or standpipes. Overall, the country’s health situation remains exceptionally fragile, as illustrated by recent outbreaks of plague (in 2014 and 2017) and measles (in 2019). Madagascar has one of the lowest levels of per capita health spending in the world, and more than three quarters of the population live in extreme poverty [31]. Still, the epidemiological transition is well underway. As a result of population ageing and changes in risk factors, the distribution of causes of death has changed considerably. In the period 1976–1980, 54% of deaths registered in Antananarivo were due to communicable, maternal, neonatal and nutritional conditions, but this proportion had dropped to 21% in 2011–2015 (Figures S2 and S3).

### Data source

This study is based on data on 249 421 residents of Antananarivo-city who died between 1976 and 2015. This corresponds to the central administrative sector of Antananarivo-Renivohitra, with a population estimated at 1.28 million inhabitants in 2018 (5% of the national population) [32]. It was not possible to reconstitute an individual database for the period before 1976 because the registers are lost.

All deaths that occur within this area should be reported to the BMH. About 60% of deaths occur at home. Relatives of the deceased contact the BMH and a physician is sent to the house of the deceased to establish a cause, based on the information provided by the family on the symptoms and circumstances preceding the death, as well as available medical documents. This is equivalent to medical certification and is different from verbal autopsy methods that have been primarily developed to identify the probable cause of death in the absence of a physician. For facility deaths, the reports are filled in by medical personnel and transmitted to the BMH by relatives. The completeness of reporting of deaths among adults (with a physician-certified cause of death) was higher than 90% in the intercensal period 1975–1993. Estimates of completeness will be updated when the detailed population counts from the 2018 census become available. In recent years, under-five mortality rates inferred from the BMH are aligned with trends derived from Demographic and Health Surveys [24]. Cause-specific mortality fractions derived from the registers are also consistent with epidemiological models [24].

The team in charge of certifying deaths currently consists of eight physicians, all of whom have been trained on the application of the International Classification of Diseases (ICD). For home deaths, an ICD-10 code is assigned based on the cause noted in plain text in the information sheet used in post-mortem interviews. For health facility deaths, the ICD code is based on the cause mentioned in the death certificate, which can come in various formats (directly with an ICD-10 code, a code from a previous revision of the ICD or reported in plain text). In the past, not all deaths in the registers had an ICD code but one physician with special training ensured that all deaths were coded in ICD-9 when registers were digitalized.

To group causes of deaths in broad categories, we used the hierarchical cause-of-death list established by the Global Burden of Disease (GBD) Study 2016 [33]. This list has four levels. The first level distinguishes between (a) communicable, maternal, neonatal, and nutritional diseases, (b) non-communicable diseases, and (c) injuries. The second level refers to 21 broad categories, such as, for example, diarrhea, lower respiratory and other common infectious diseases among one group of causes. The third and fourth levels refer to more detailed causes of death, such as, for example, intestinal infectious diseases (level 3) and typhoid fever (level 4). For this study, we considered only the second level of the GBD hierarchy. All ICD 9 codes were mapped to a GBD cause of death. Some ICD codes were considered ‘garbage codes’. This refers not only to causes identified as ‘undefined’ in the specific ICD chapters, but also deaths attributed to causes which should not be considered as initial causes, such as dehydration or septicemia. We used a simplified redistribution algorithm to map these codes to acceptable GBD causes. For example, ill-defined cardiovascular diseases were redistributed to ischemic heart disease and other cardiovascular and circulatory diseases. The redistribution is summarized in the Appendix and described in detail elsewhere [24]. We conducted a sensitivity analysis using ICD-9 chapters before any redistribution of garbage codes, and results were similar to those obtained with the GBD cause categories (Appendix).

### Statistical analyses

Mortality rate ratios associated with months are obtained from a Generalized Additive Mixed Model using a Negative Binomial distribution, a generalization of the Poisson distribution that accounts for overdispersion. The model includes a penalized regression spline [34] to model long-term trends in mortality and avoid over-fitting, month as a random effect to model seasonality and year as a random slope to assess any change in seasonality, after stratifying by age groups. Age groups consisted of infants (less than 1 year old), young children (1 to 4 years old), older children and young adults (5- to 59-year-olds) and people aged 60 or above. We used the Bayesian (BIC) Information Criteria to choose the best model among a model with penalized splines for the trend only (model 1); a model with random intercepts for months (model 2); and a model with random slopes and random intercepts (model 3). Retaining models that minimized the BIC with a difference of more than 10 [35] allowed us to characterize first, if seasonality was present (model 2) and second if it was changing over time (model 3). Because of the unequal number of days in a month, we multiplied each monthly death count by 30.4 and divided by the number of days in each month. The full model (model 3) can be expressed as follows:

$$y_{t}∼NB\left(\alpha_{j\left[i\right]}+\beta_{j\left[i\right]}Year_{i}+s\left(t_{i}\right)\right)$$

$$\left(\begin{array}{c} \alpha_{j}\\ \beta_{j} \end{array}\right)∼N\left(\left(\begin{array}{c} \mu_{\alpha }\\ \mu_{\beta } \end{array}\right),\left(\begin{array}{cc} \sigma_{\alpha }^{2} & \rho_{\sigma_{\alpha }\sigma_{\beta }}\\ \rho_{\sigma_{\alpha }\sigma_{\beta }} & \sigma_{\beta }^{2} \end{array}\right)\right)$$

where $y_{t}$ are monthly counts of deaths, s() is a penalized spline, *j* ∈ {Jan., Feb., …, Dec.}, $Year_{i}\in \left\{-20,-19,\ldots 0,1,\ldots 19,20\right\}$ where 0 represents 1996, the middle of the analysis period and *t* ∈ {1, 2, ..., 480} is a continuous variable reflecting the count of month.

We evaluated goodness-of-fit by visual inspection of the deviance residuals, considering that a good fit was obtained if 95% of the deviance residuals were between −2 and 2 standard deviations and no large outliers were present. All analyses were conducted using R statistical software.

## Results

Out of the 249,421 notified deaths over the 40 years studied, 37,775 (15%), 35,138 (14%), 101,111 (41%) and 75,266 (30%) consisted of infants, children aged 1–4, older children and adults aged 5–59, and the population aged 60 or above, respectively (the date of birth or date of death was missing for 131 cases).

**Figure 2.**
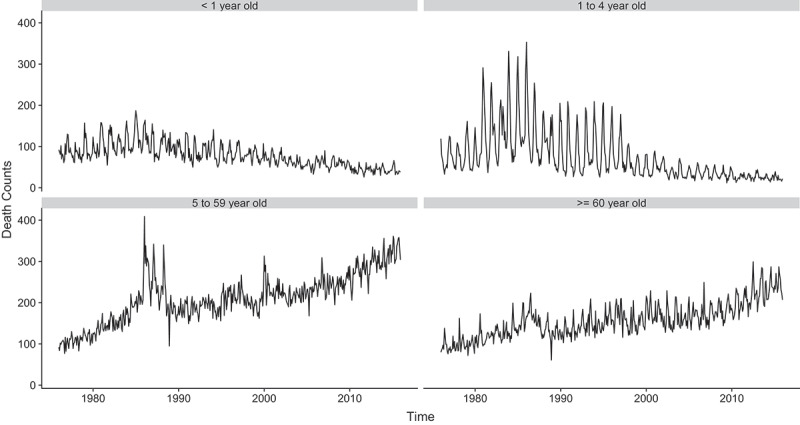
Monthly mortality counts by age group, over time

Due to shifts in the age structure of the population and the decline in under-five mortality, the two younger age groups experienced a downward trend while the two older age groups showed an upward trend in their monthly mortality counts (Figure 2). The regular ups and downs in these time series correspond to the seasonal variations. The amplitude of these monthly variations seems to have reduced over time for infants and young children.

### Seasonal patterns of all-cause mortality

We used seasonality plots to visually examine seasonality (Figure 3). We divided the analysis period into three periods of equal length (13 to 14 years) to evaluate if seasonality was changing over time. Figure 3(a,b) highlight the seasonality in infant and child mortality. This is easily spotted from the U-shape of monthly mortality counts. Overall, 60% of all under-five deaths occurred in the hot and rainy season from November to April. Seasonality seems to have reduced in recent years. Considering older children and adults aged 5–59, no regular pattern in mortality was observed on a yearly basis. Mortality of the population aged 60 and above exhibited seasonality with the dry season characterized by higher death counts.

**Figure 3.**
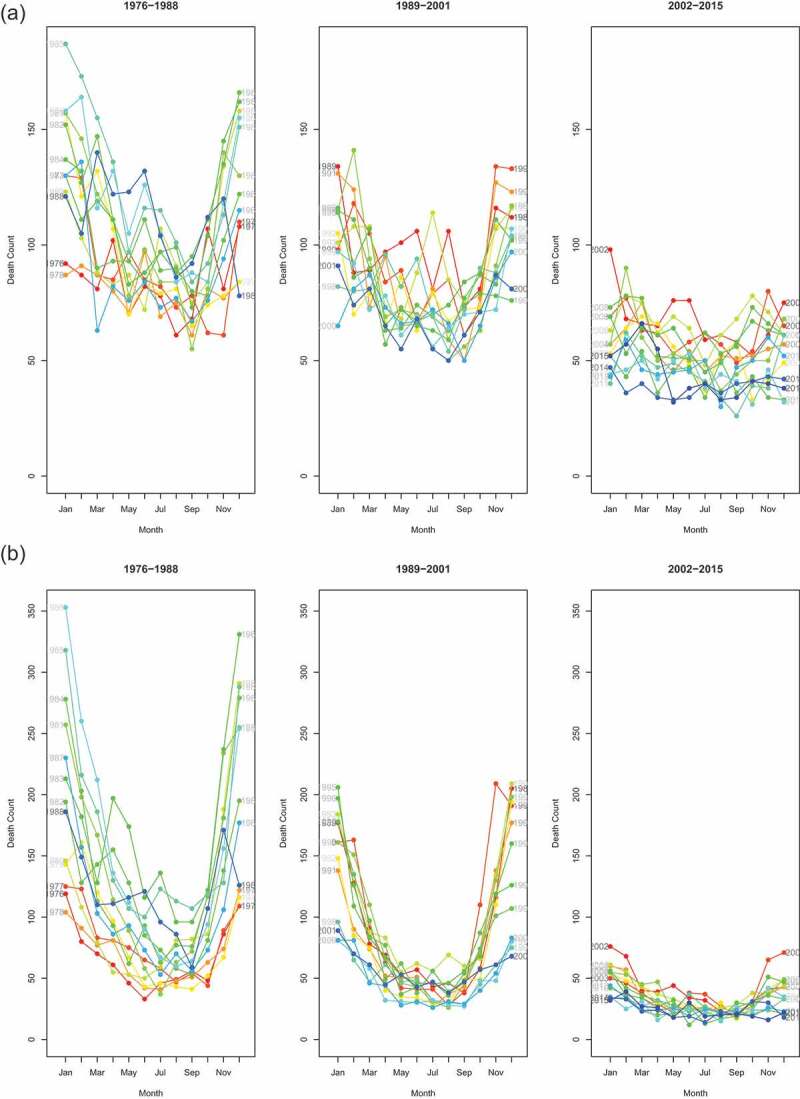
Seasonality plots, by age groups, Antananarivo (1976–2015). In each series, red curves refer to the earliest years and blue curves to the latest years

**Figure 3.**
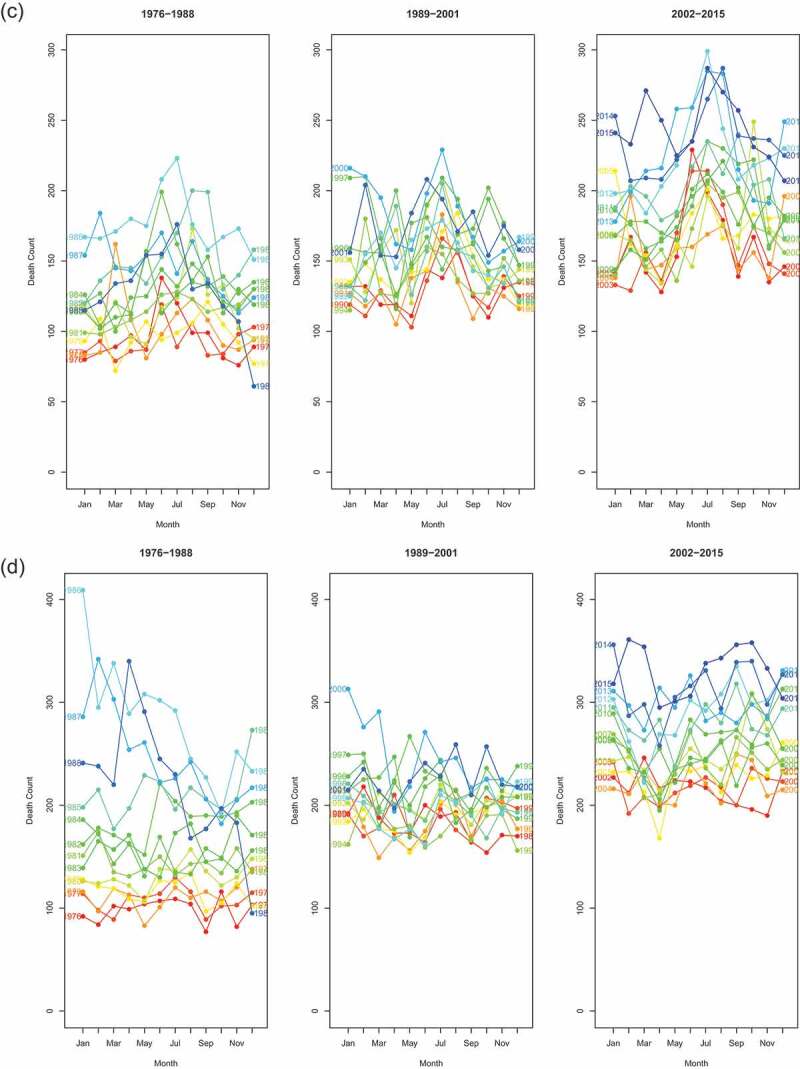
(Continued)

Generalized Additive Mixed regression models (GAMM) allow us to test if seasonality was present and changed over time. Comparing our models according to the BIC (Appendix A.1), only the age group of children aged 1 to 5 experienced changes in the seasonality of all-cause mortality. That is, the full model (model 3) had a lower BIC than model 2 (with random intercepts only) and the difference was larger than 10. Among infants and people aged 60 and above, model 2 provided the best fit according to our criteria, suggesting that they experienced a constant seasonality over the analysis period. However, when considering infants aged less than 1, the model allowing for changes in seasonality (model 3) came close to outperforming the model reflecting constant seasonality. In contrast, mortality of adults aged 5–59 was best modelled by a simple penalized spline (model 1), suggesting that seasonality of all-cause mortality was not observed for this age group. Results were similar when we subdivided this large age group into smaller age segments (5–14, 15–29, 30–44 and 45–60 years-old) (Appendix). We present in Figure 4 the random coefficients associated with the full model (model 3) allowing for changing seasonality in all age groups, despite the fact that it does not always provide the best fit. We do this in order to be able to compare random intercepts across age groups, and because the fact that some random slopes are significant is informative. As we assumed a Negative Binomial distribution, the results are expressed in terms of rate ratios (RRs) and refer to the mid-period (1996). Random intercepts thus reflect the RR of mortality in that month at the mid-period (1996), while random slopes capture how much this is changed over the time-course – e.g. a slope that is significantly less than 0 indicates that the RR for that month declines by each year moved forwards in the time-series; and conversely, a RR significantly greater than 0 indicates that the RR for that month increases relative to the mid-period.

**Figure 4.**
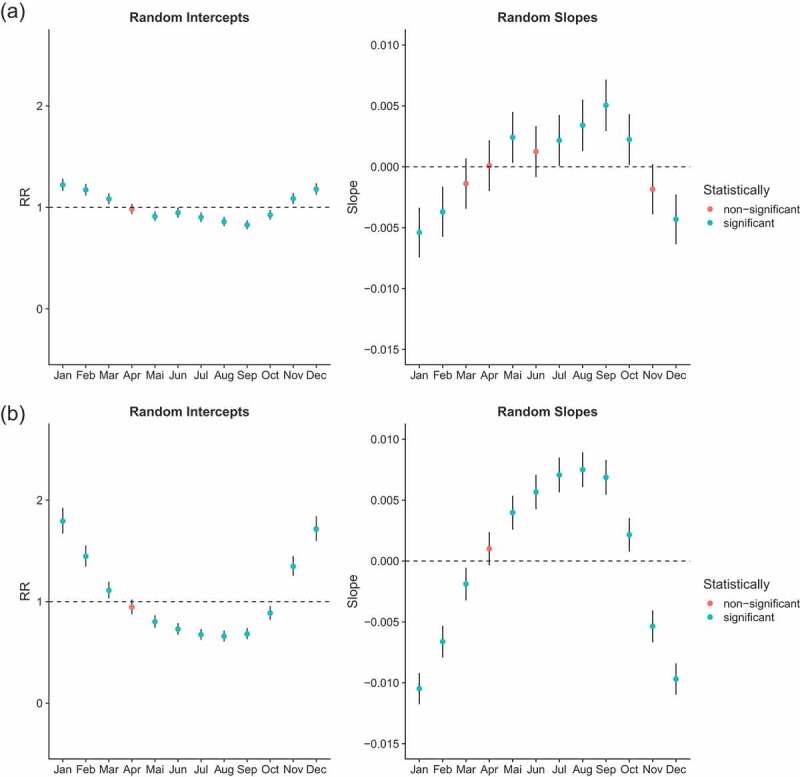
Mortality rate ratios from a NB GAM model (model 3) stratified by age group (rows); left-hand column (‘random intercepts’) indicates rate ratios for mortality in each month at the mid-period, and the right-hand column (‘random slopes’) shows how each changes RR with advancing years in the time-series, i.e. values less than 0 indicates declines in that month’s RR. Note: different scales are used for the y-axis. Scales for Figure 2(a,b) are the same. Scales for Figure 2(c,d) are the same for random intercepts but different for random slopes

**Figure 4.**
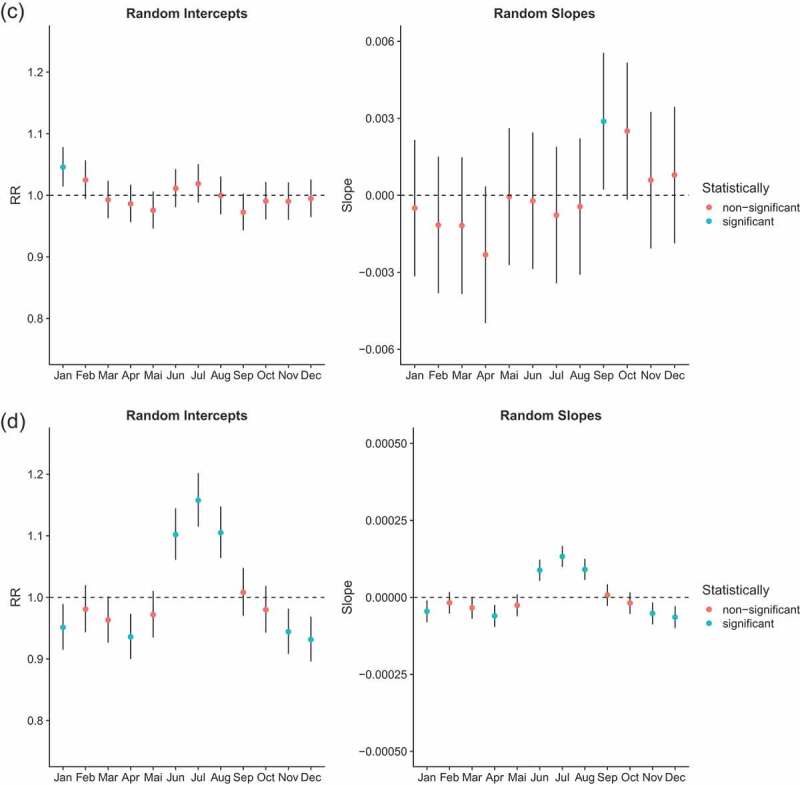
(Continued)

Month effects for infants reflect a slight U-shape pattern (Figure 4(a)). Five months have RRs that are significantly higher than the average monthly rate, and 6 months have RRs that are significantly lower. In 1996, the higher mortality RRs were January and December, estimated at 1.22 (95% CI: 1.16–1.28) and 1.18 (95% CI: 1.12–1.24), respectively. The lower RR was estimated for September with 0.83 (95% CI: 0.78–0.87). Because random slopes have a pattern opposite to random effects, the excess mortality observed in some months (especially November to February) decreases over time, while the lower mortality rates in the months of May to September gradually converge to the annual average. However, we reiterate that the full model does not outperform model 2 (with random intercepts only), even though some random slopes are statistically different from 0.

Random intercepts for young children (1–4 years old) exhibit a clear U-shape with mortality RRs reaching 1.79 (95% CI: 1.67–1.92), 1.44 (95% CI: 1.34–1.55), 1.35 (95% CI: 1.25–1.45) and 1.72 (95% CI: 1.60–1.84) for January, February, November and December 1996, respectively. Mortality rates estimated in December are more than twice those estimated in September, as compared with 1.18 in infants. The random slopes show the exact reverse trend than that observed for the random intercepts (comparing the right side and left side of Figure 4(b)). Months that are characterized by a high RR have statistically significant negative slopes and vice versa. In other words, the difference of RR between months reduces over time.

Mortality among children aged 5 years and above and adults aged less than 60 does not reflect any seasonal pattern. All months (except January) show RRs that are not statistically significantly different from one (Figure 4(c)). This is in line with what has been observed in Figure 3(c).

The seasonality of mortality for the oldest age group is characterized by a slight inverse U-shape. It is the reverse pattern of what was observed in children (also note the change of scales). The RRs for June, July and August 1996 were 1.10 (95% CI: 1.06–1.15), 1.16 (95% CI: 1.11–1.20) and 1.11 (95% CI: 1.06–1.17), respectively. Random slopes for June, July and August suggest that the peak associated with dry season has increased over time, although the full model with changing seasonality does not outperform the reduced one.

### Seasonal patterns of major causes of deaths

We defined major causes of death as GBD causes consisting of more than 5% of deaths of a given age group experiencing seasonality in the previous section. We then ran the three different models, again using BIC to select the best model (Table 1).

**Table 1.** Seasonality of major causes of death, by age groups

Values inside the cells are BIC values associated to each major cause, age group and model.

**Figure 5.**
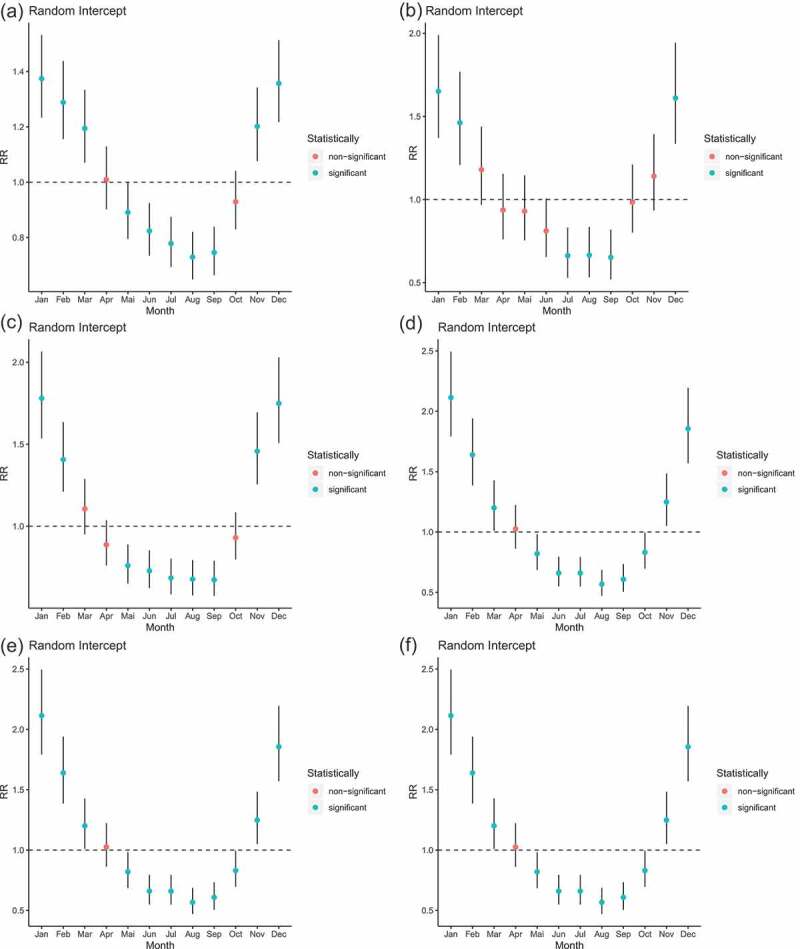
Mortality rate ratios from a NB GAM model (model 2), stratified by major cause and age group; ‘random intercepts’ indicates rate ratios for mortality in each month at the mid-period. Note: different scales are used for the y-axis

None of the major causes of death showed varying seasonality over time (i.e. model 3 was never selected using BIC). Four others did not show any seasonality (model 2 was not selected). These were: neonatal disorders and other non-communicable diseases (congenital birth defects) for children aged less than 1 year, and diarrhea, lower respiratory, and other common infectious diseases and neoplasms for people aged 60 or above. Six major causes exhibited constant seasonality. Figure 5 displays the monthly random effects (from model 2) associated with these causes of death.

For infants, diarrhea, lower respiratory, and other common infectious diseases experience a U-shape in mortality rates, with higher and lower RRs during the rainy and dry season, respectively. The same is true for nutritional deficiencies in this age group. The maximum RRs are attained in January and December and are 1.36 (95% CI: 1.23–1.50) and 1.33 (95% CI: 1.21–1.47) for diarrhea, lower respiratory, and other common infectious diseases, and 1.63 (95% CI: 1.37–1.95), and 1.54 (95% CI: 1.29–1.84) for nutritional deficiencies. The minimum RRs are attained in August and September 1996 and are estimated at 0.75 (95% CI: 0.67–0.83) and 0.74 (95% CI: 0.67–0.82) for diarrhea, lower respiratory, and other common infectious diseases. Months exhibiting the lowest RRs were July and September 1996 for nutritional deficiencies with 0.67 (95% CI: 0.57–0.83) and 0.65 (95% CI: 0.52–0.81).

Considering children aged 1 to 5, seasonality of mortality associated with these two broad causes of death (the group of diarrhea, lower respiratory, and other common infectious diseases, and the group of nutritional deficiencies) also exhibit this pattern but with a larger amplitude. The difference between RRs of adjacent months is greater than for children less than 1 year old. RRs for nutritional deficiencies reach their higher values at 2.09 (95% CI: 1.77–2.46) and 1.84 (95% CI: 1.59–2.17) in January and December 1996, respectively.

For people aged 60 or above, cardiovascular diseases have coefficients for June, July, August and September that are 1.11 (95% CI: 1.06–1.17), 1.19 (95% CI: 1.13–1.25), 1.14 (95% CI: 1.08–1.20) and 1.05 (95% CI: 1.01–1.11), respectively. Diabetes, urogenital, blood and endocrine diseases have a statistically significant RR in July reaching 1.22 (95% CI: 1.12–1.33).

## Discussion

We observed strong seasonality of mortality in Antananarivo, especially among children under age 5 and the elderly.

In infants, the seasonality of deaths is dominated by the association between hot temperatures and rainfall, and two groups of causes: (1) diarrhea, lower respiratory, and other common infectious diseases, and (2) nutritional deficiencies. These two groups of causes are closely intertwined, as malnutrition will make diarrhea and other infectious diseases such as pneumonia or measles more lethal and these infections will in turn exacerbate child malnutrition [36]. Together, these two categories of causes accounted for 48% of deaths below age 1 in 1976, but this percentage declined to 36% in 2015, as a result of the epidemiological transition. Conversely, the percentage of deaths due to neonatal disorders and other non-communicable diseases (mostly congenital birth defects) increased from 46% to 54% in the period 1976–2015. As mortality rates from these two last groups of causes do not exhibit seasonality, the shift in disease patterns in infant mortality explains why there is some indication that seasonality is declining in this age group. Random slopes associated with years in our all-cause mortality model were negatively correlated with random effects. However, the changes over time are modest, and overall, a model assuming a constant seasonality in infant mortality is the one to be preferred statistically.

Childhood mortality (1–4 years) seems more sensitive to environmental factors than infant mortality as we observe here particularly strong seasonal patterns at these ages. As in infants, seasonality of all-cause mortality in this age group is largely due to two sets of causes: (1) diarrhea, lower respiratory and other common infectious diseases, and (2) nutritional deficiencies. Taken together, these two sets of causes accounted for 89% of child deaths in 1976 and 58% in 2015. As a result, monthly variation is more pronounced than in infants, and changes over time in the seasonality are faster. Overall, the seasonality of all-cause childhood mortality has attenuated. Again, this is driven by the reduction in the burden of these two causes, rather than changes in the seasonality of mortality attributable to these causes.

The excess infant and child mortality observed at the peak of the rainy season can be associated with seasonal food shortages and increased pathogen circulation. As indicated earlier, the lean season is characterized by substitution of secondary foods and compression in overall food consumption. In the capital city, the price of rice in December and January is about 17% higher than in June [25]. Such fluctuations could contribute to a deterioration of children’s nutritional status in the lean season, as has been observed among adult women in the Amoron’i Mania Region of Madagascar [37]. As regards increased circulation of pathogens, a 32-year investigation of the quality of drinking water in Antananarivo showed that cumulative rainfall was associated with higher contamination by coliform bacteria, especially in January and February, due to the overload of the filtration system [38]. Peaks of infections with noroviruses in children with acute gastroenteritis have been observed in the city in November and December [39]. A study on the viral etiology of influenza-like illnesses in Antananarivo showed a distinct seasonality of infections from human respiratory syncytial virus (RSV), influenza virus A (FLUAV) and coronavirus OC43, but peaks were rather located at the start and end of the rainy season (September–October and March–April) [40].

In adults aged 60 and above, seasonal patterns in all-cause mortality observed in this study are in line with those of deaths from cardiovascular diseases and a group of causes encompassing diabetes, urogenital, blood and endocrine diseases. Most deaths from this second group were assigned to diabetes mellitus and chronic kidney disease. Thermal stress can represent a challenge for individuals with diabetes, as it disrupts homeostasis, especially for the cardiovascular system and glycaemia. The literature on the effects of extreme temperature on morbidity and mortality in individuals with diabetes has mostly highlighted heightened risks of dying in case of heat stress [41,42], with less evidence of an association between cold stress and excess mortality. It is possible that the winter peak observed in Antananarivo reflects difficulties experienced by some physicians in discerning the actual cause of death in diabetics. They might have registered diabetes mellitus as the underlying cause of death, while the death was in fact due to cardiovascular diseases, for which diabetes is a prime risk factor. However, cold temperatures have been shown to increase the risk of diabetes mortality in China [43], and peaks in diabetes mortality in winter months have also been observed in the Netherlands [5] and Japan [14]. Hence, the excess mortality from diabetes in the winter months could be genuine. The percentage of deaths due to diabetes, urogenital, blood and endocrine diseases increased slightly (but not significantly) from 9% in 1976 to 11% in 2015. The percentage of deaths due to cardiovascular diseases increased significantly over the period, from 46% in 1976 to 53% in 2015 among the elderly. This reflects the normal path of the epidemiological transition, and is also associated with aging of the population. This pattern explains why random slopes were positively correlated with random effects in this age group, suggesting that seasonality in all-cause mortality has accentuated in older adults. Yet changes over time have been modest and when comparing models using BIC, the model with constant seasonality is to be preferred, as is the case for infant mortality.

Our study has some limitations. First, we are not able to accurately separate the effects of different seasonal factors (biological or behavioral) on mortality. Second, we do not include data on temperature and rainfall in our models. Third, biases could arise due to our redistribution of some deaths in GBD cause categories. However, we accounted for the seasonality in our redistribution, and when we run the same models on groups of ICD-9 codes before any redistribution, we obtained similar results (Appendix). Finally, this analysis was restricted to residents of Antananarivo-city, identified from a question on the address of the deceased. Nevertheless, no question was asked about the length of residence of the deceased, and it is possible that some people registered as residents in the database were recent migrants, including migrants who may have settled in the city to seek medical treatment. Conversely, some residents of Antananarivo may have died outside the city while migrating – including those seeking supportive care in their home community. Recent research in Burkina Faso suggests migrations out of Ouagadougou from terminally ill adults were more likely when deaths were due to non-communicable diseases, compared to communicable diseases [44]. They may have more time to travel than those affected by acute diseases or diseases requiring intensive care. Migrations at the end of life could introduce small biases in the cause-specific mortality estimates, but they are unlikely to have a large impact on our results related to the seasonality of deaths.

This study should be prolonged by examining some specific causes, such as measles. In a context of high vulnerability to extreme weather events, the database could also be used to study associations between temperature and rainfall and peaks in some cause-specific mortality in order to inform early warning systems. The association between air pollution, a major health issue in Antananarivo, and the seasonality of deaths is another area for future research. Finally, monthly variations in mortality should be analyzed outside of the capital city. There are large variations in climate, malaria endemicity and vaccination coverage across the country. Systems of death notification are also in place in the the other six large urban centers (Tamatave, Antsirabé, Fianarantsoa, Mahajanga, Tuléar, Diego-Suarez), but the municipal offices of hygiene are not all equally well-staffed and equipped, and the completeness of death registration in these cities remains unknown. Outside of these urban centers, information on causes of death mainly comes from health facilities statistics, thus excluding community deaths. In addition, the annual yearbook of health statistics does not report on causes of death classified according to the ICD [45].

## Conclusions

This study illustrates the value of local death notification systems for health planning, in a country where the civil registration system is deficient at the national level. We showed that there is a pronounced seasonality of mortality among young children and the elderly in Antananarivo, and identified the causes of death that contribute most to these seasonal variations. These results could contribute to health programming, to schedule vaccination campaigns or awareness campaigns for the population.

## Supplementary Material

Supplemental Material

Supplemental Material

Supplemental Material

Supplemental Material

## Data Availability

The dataset containing ICD-9 codes and GBD cause categories needed to replicate this analysis are available upon request.

## Paper context

The seasonality of cause-specific mortality is poorly documented in sub-Saharan Africa, due to incomplete registration of deaths. Our study takes advantage of a system of death notification in Antananarivo, and shows large monthly variations in mortality among children and the elderly, with two opposite patterns in these age groups. Seasonality has decreased among children due to a reorganization of the hierarchy of causes of death. Health planning could be improved by taking into account seasonality.
