# Supplementary figures and images for: Long-term trends in seasonality of mortality in urban Madagascar: the role of the epidemiological transition
Source: Glob Health Action. 2020 Feb 6;13(1):1717411. doi: 10.1080/16549716.2020.1717411 (PMC7034494; doi:10.1080/16549716.2020.1717411)

Normal Q-Q Plot

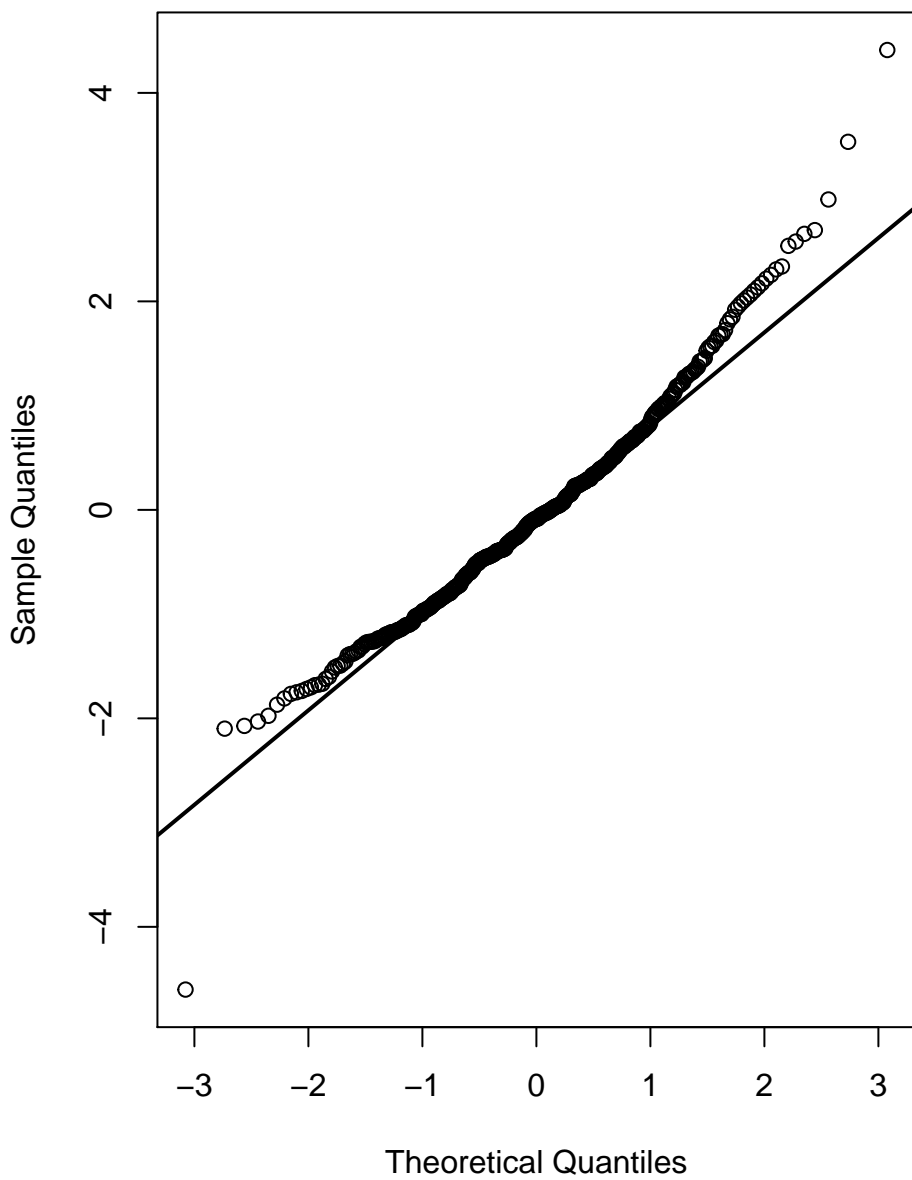

[60,Inf)

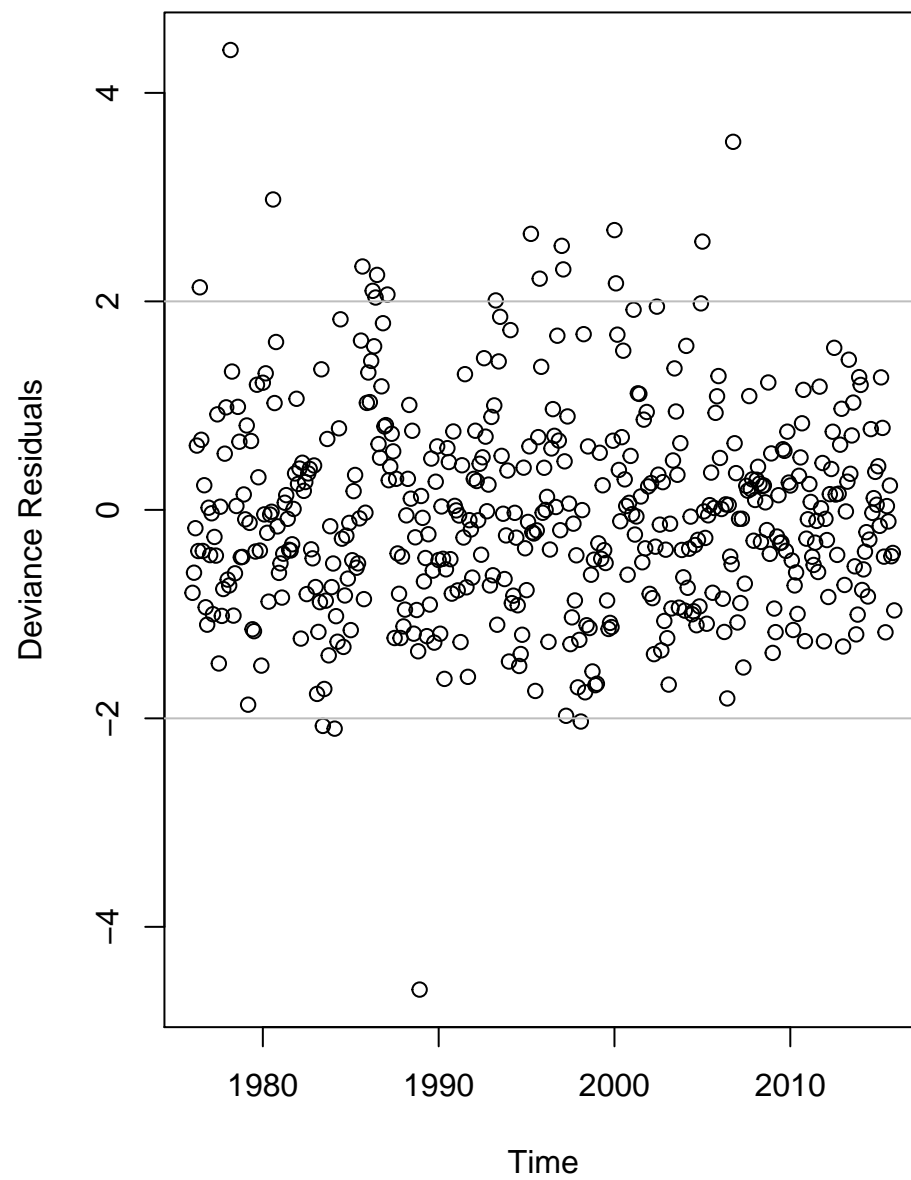

Supplement: Supplemental Material [file ZGHA_A_1717411_SM8723.pdf]

Normal Q-Q Plot

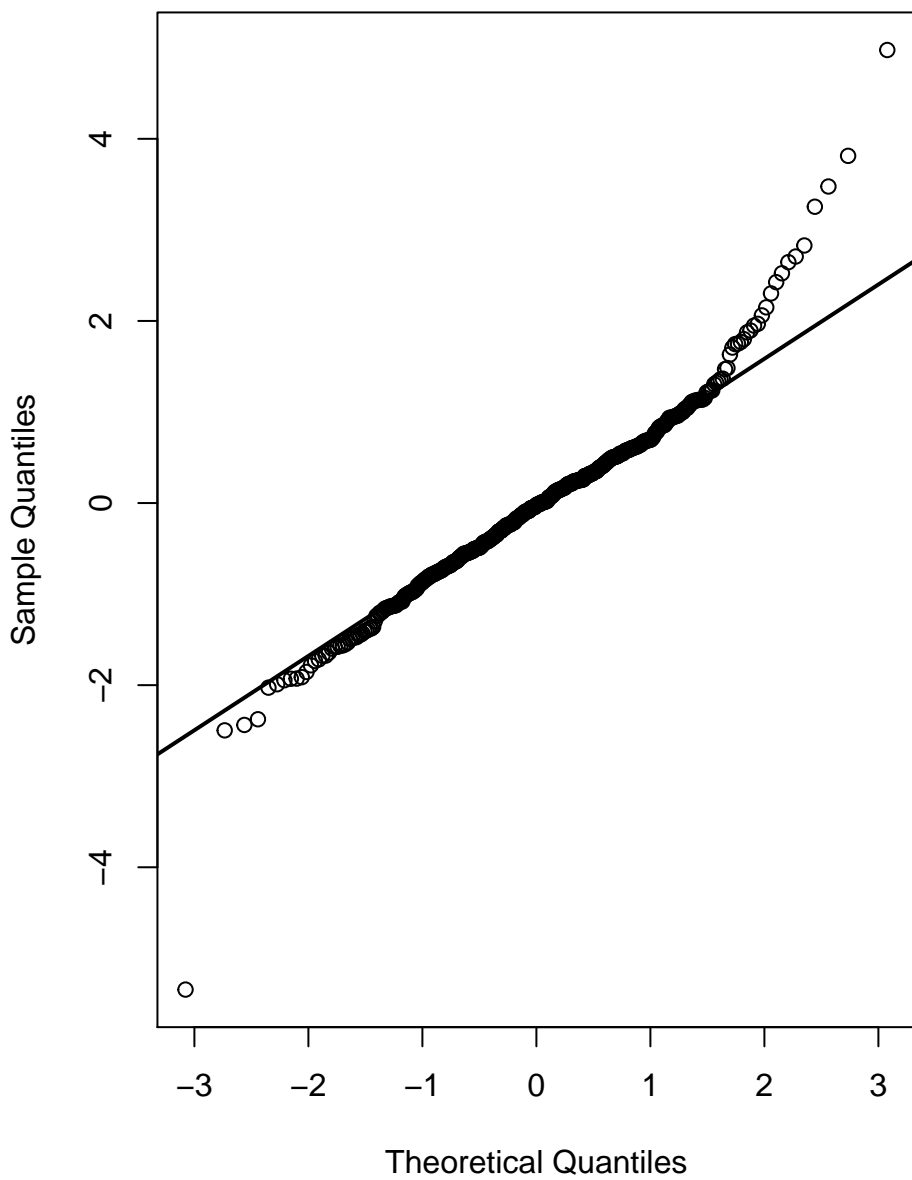

[5,60)

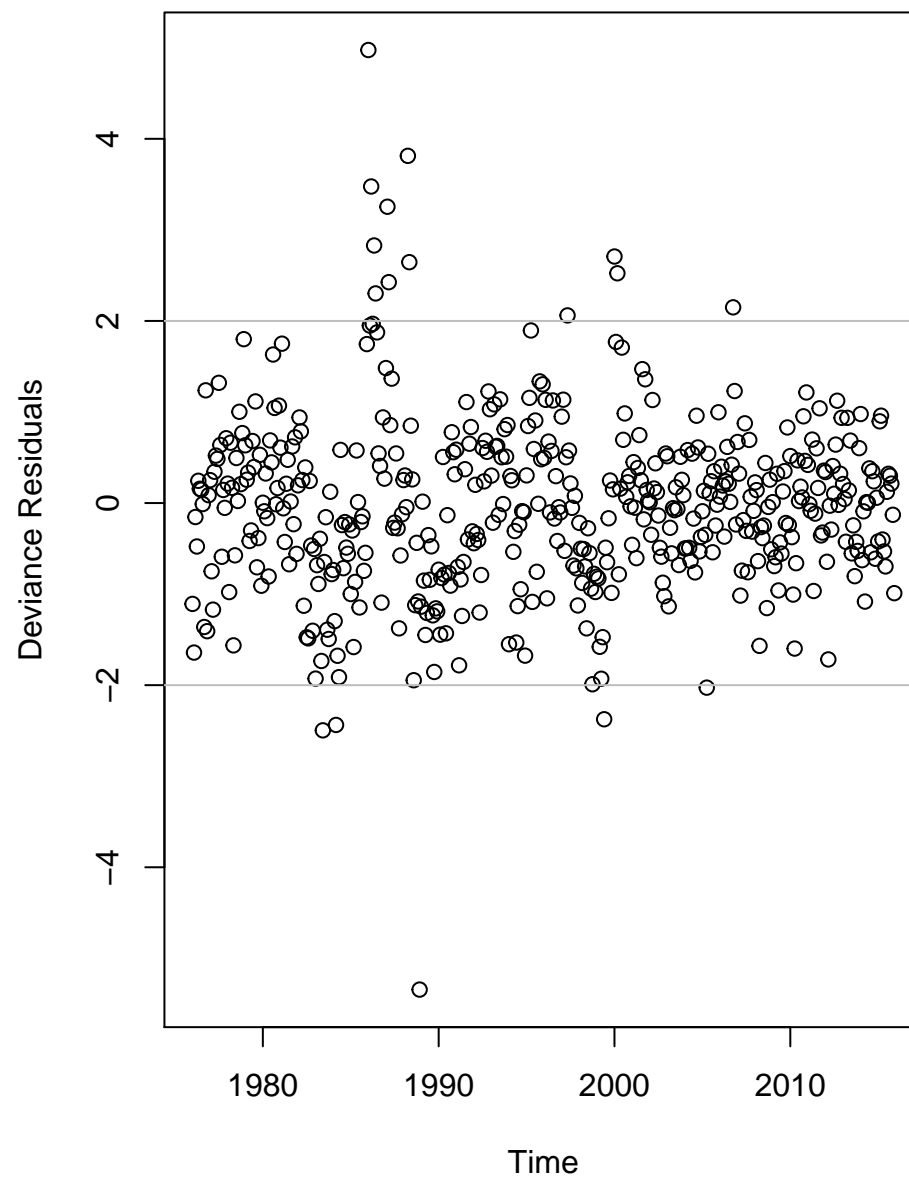

Supplement: Supplemental Material [file ZGHA_A_1717411_SM8722.pdf]

Normal Q-Q Plot

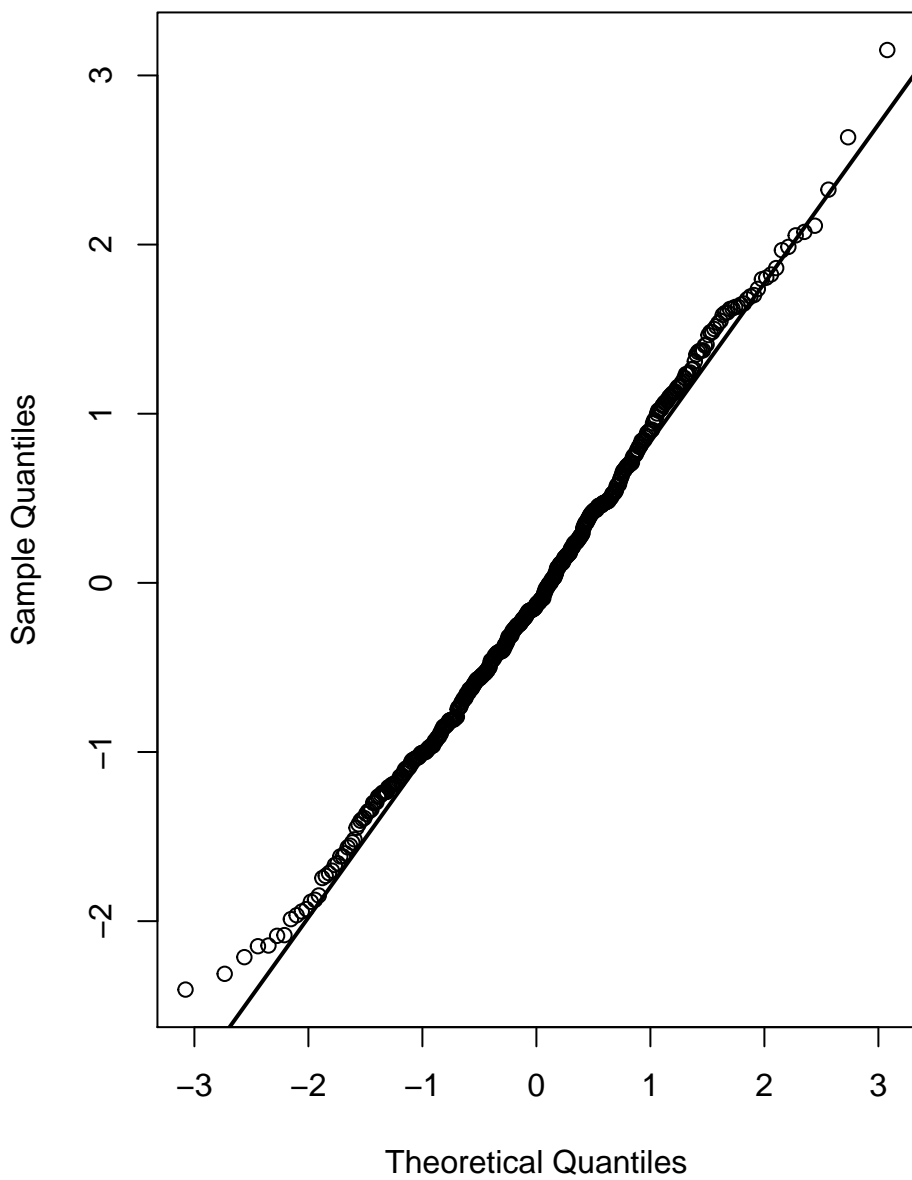

[1,5)

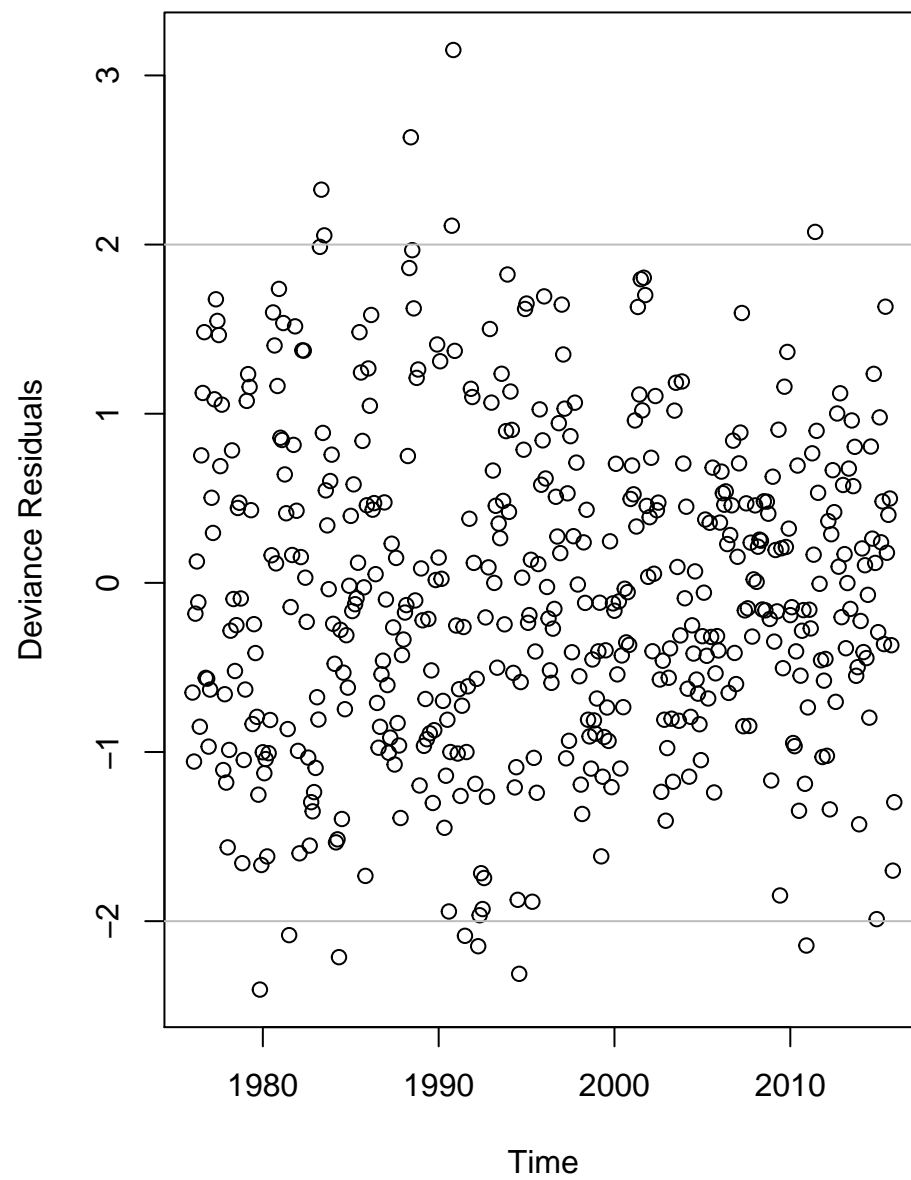

Supplement: Supplemental Material [file ZGHA_A_1717411_SM8720.pdf]

Normal Q-Q Plot

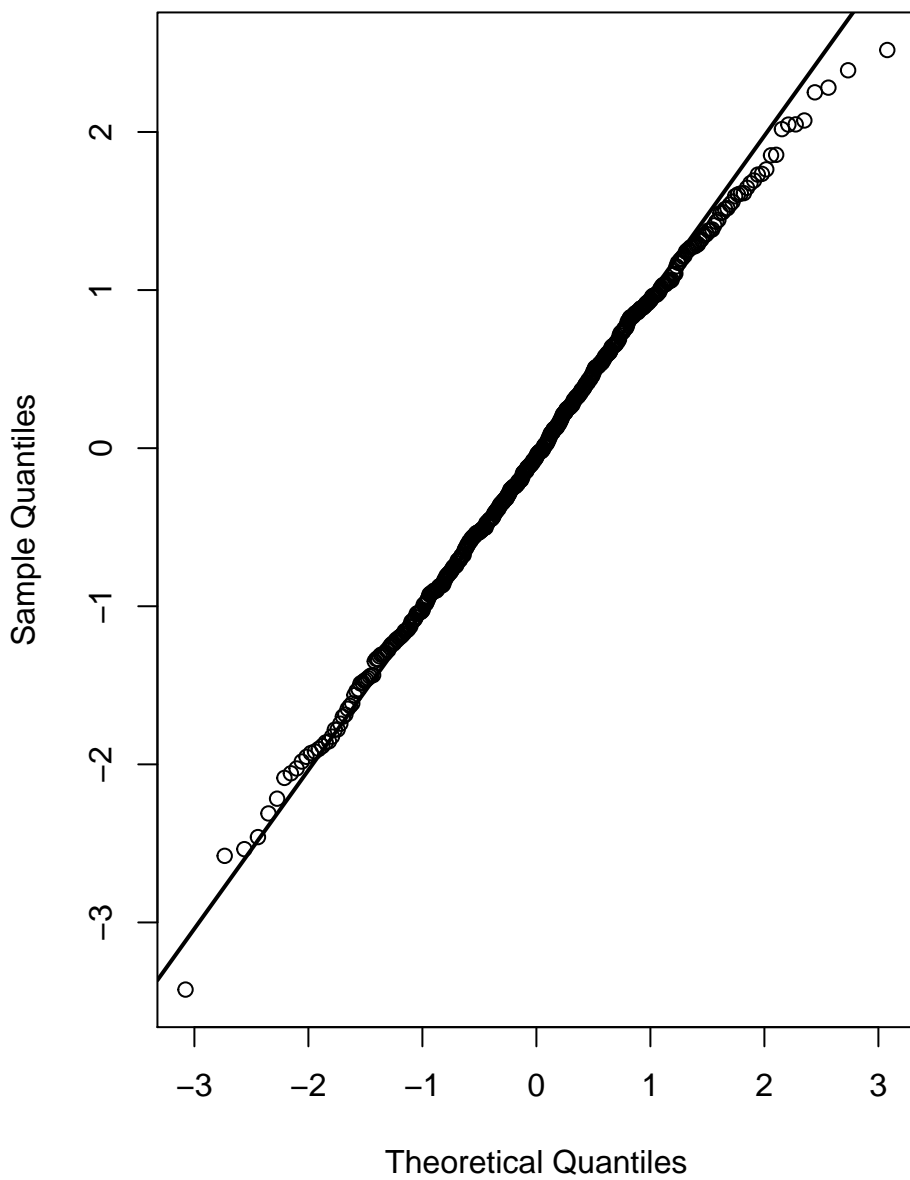

[0,1)

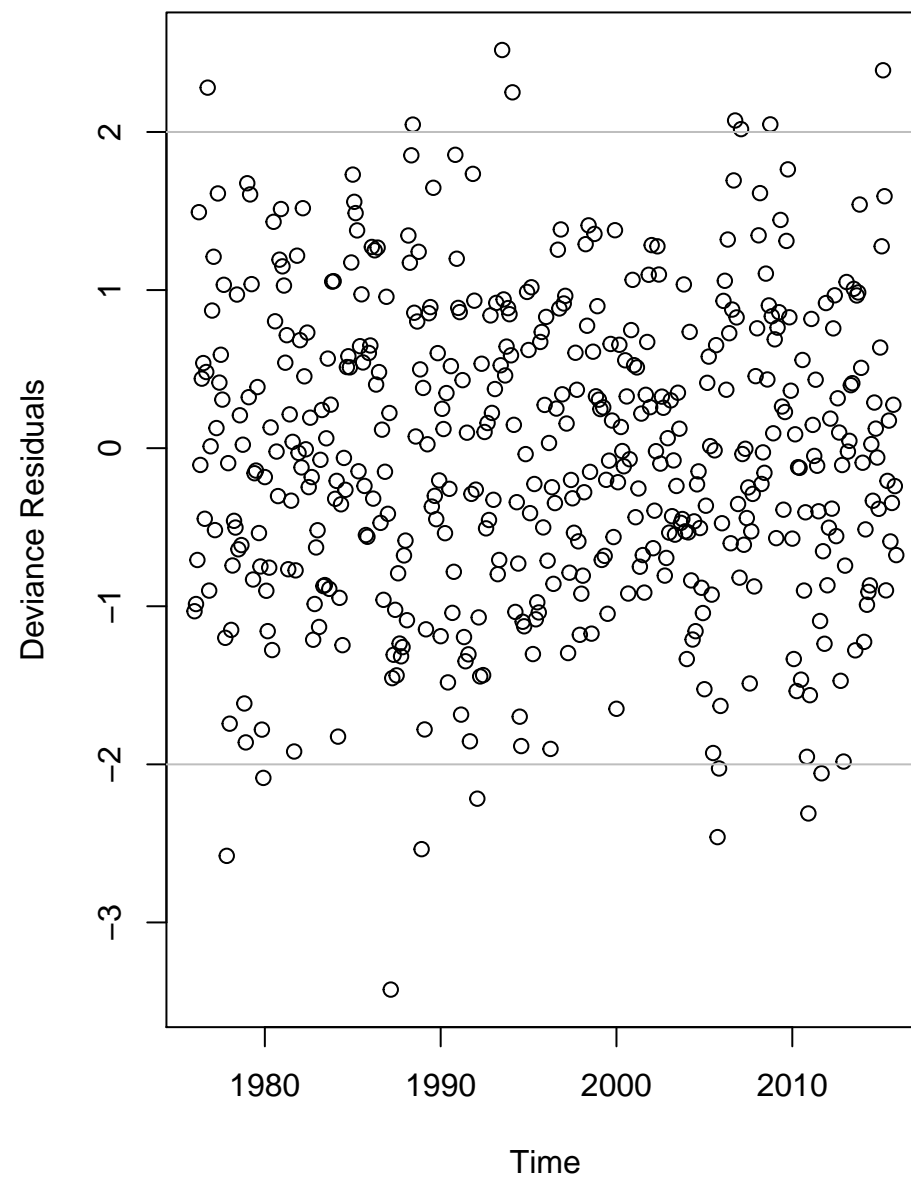

Supplement: Supplemental Material [file ZGHA_A_1717411_SM8717.pdf]
